# Pregnancy outcomes among women born in Somalia and Sweden giving birth in the Stockholm area – a population-based study

**DOI:** 10.1080/16549716.2020.1794107

**Published:** 2020-08-03

**Authors:** Anna Akselsson, Helena Lindgren, Susanne Georgsson, Karin Pettersson, Viktor Skokic, Ingela Rådestad

**Affiliations:** a Department of Health Promoting Science, Sophiahemmet University, Stockholm, Sweden; b Department of Women and Children’s Health, Karolinska Institutet, Stockholm, Sweden; c The Swedish Red Cross University College, Stockholm, Sweden; d Department of Clinical Science, Intervention and Technology, Karolinska Institutet, Stockholm, Sweden; e Institute of Clinical Sciences, Sahlgrenska Academy, University of Gothenburg, Gothenburg, Sweden

**Keywords:** Fetal movements, Mindfetalness, awareness of fetal movements, Apgar score, stillbirth

## Abstract

**Background:**

Studies report that women born in some African countries, after migrating to the Nordic countries, have worse pregnancy outcomes than women born in the receiving countries. With the aim of identifying unmet needs among Somali-born women, we here study this subgroup.

**Objective:**

We compared pregnancy outcomes among women born in Somalia to women born in Sweden. Further, we investigated whether the proactive maternal observation of fetal movements has effects on birth outcomes among women born in Somalia.

**Methods:**

In Stockholm, half of the maternity clinics were randomized to intervention, in which midwives were instructed to be proactive towards women by promoting daily self-monitoring of fetal movements. Data for 623 women born in Somalia and 26 485 born in Sweden were collected from a population-based register.

**Results:**

An Apgar score below 7 (with stillbirth counting as 0) at 5 minutes was more frequent in babies of women born in Somalia as compared to babies of women born in Sweden (RR 2.17, 95% CI 1.25–3.77). Babies born small for gestational age were more common among women born in Somalia (RR 2.22, CI 1.88–2.61), as were babies born after 41 + 6 gestational weeks (RR 1.65, CI 1.29–2.12). Somali-born women less often contacted obstetric care for decreased fetal movements than did Swedish-born women (RR 0.19, CI 0.08–0.36). The differences between women born in Somalia and women born in Sweden were somewhat lower (not statistically significant) among women allocated to proactivity as compared to the Routine-care group.

**Conclusions:**

A higher risk of a negative outcome for mother and baby is seen among women born in Somalia compared to women born in Sweden. We suggest it may be worthwhile to investigate whether a Somali-adapted intervention with proactivity concerning self-monitoring of fetal movements may improve pregnancy outcomes in this migrant population.

## Background

Women who have migrated to western industrialized countries have a higher risk of adverse pregnancy outcomes than receiving-country nationals [1,2]. One such risk group is Somali-born women having migrated to Sweden [3,4]. A meta-analysis [4] showed that women from Somalia, in comparison to women born in Sweden, had a higher risk of giving birth to a child with an Apgar score of below seven at 5 min after birth. Sub-standard care, lack of communication, and worse pre-pregnancy health have been suggested as explanations for the differences in outcomes [5,6]. According to a report published in *The Lancet*, sub-standard care contributes to 20–30% of all stillbirths in the world [7]. It may be worthwhile to identify and, by extension, find means to address unmet needs in migrant women enrolled in maternity care in Sweden.

In Sweden, with its 10 million inhabitants, 18.5% of the population were born in a country outside Sweden [3] and, in 2017, 66 369 Somali lived in Sweden, of which 37% were women of childbearing age (WHO definition: 15–49 years) [8]. Moreover, one-fifth (11 905) of the Somali in Sweden lived in Stockholm [3]. The Swedish National Board of Health and Welfare recently published a report presenting risk factors for stillbirths in Sweden, and being born in Africa, particularly sub-Saharan Africa was highlighted [9]. Maternity care in Sweden provides a great opportunity to prevent negative outcomes and to adapt and tailor care, when needed, for certain groups of women. The Hooyo-project is one ongoing study for improving antenatal care for women from Somalia with a focus on group antenatal care [10].

Decreased fetal movements are associated with negative pregnancy outcomes such as fetal growth restriction and stillbirth [11–14]. It has been suggested that shortening the time from when a woman experiences decreased fetal movements until she seeks care may improve the pregnancy outcome [15]. To investigate whether the proactivity of midwives toward pregnant women in promoting daily self-monitoring of fetal movements (Mindfetalness) improves pregnancy outcomes, we performed a cluster-randomized controlled trial in the Stockholm area [16,17]. No differences were seen in Apgar score of less than seven at 5 min, but few babies were born small for gestational age and in need of neonatal care in the Mindfetalness group. We also observed that women allocated to monitor fetal movements more often had a spontaneous start of labor and a lower frequency of cesarean sections. Women in the Mindfetalness group contacted health care often due to decreased fetal movements than women in the routine-care group.

With the overall aim of identifying the needs of Somali-born pregnant women, we performed a sub-analysis in the Mindfetalness project. Here we study the pregnancy outcomes of Somali-born women as compared to Swedish-born women among a small sub-group of the women who were randomly allocated. We were also interested in determining the extent to which they contacted obstetric care for decreased fetal movements. Notwithstanding the problems with precision in this small subgroup, and that new validity problems may emerge when such a subgroup is demarcated, we also wanted to investigate whether it was possible to determine if the effects of proactivity that we saw among all of the included women were larger or smaller among Somali-born women.

## Methods

Details of the study are published elsewhere [16,18]. In short, pregnant women registered at a maternity clinic in Stockholm in Sweden were randomized either to be informed by their midwife about a self-monitoring method for observing fetal movements or to routine care.

**Figure 1.**
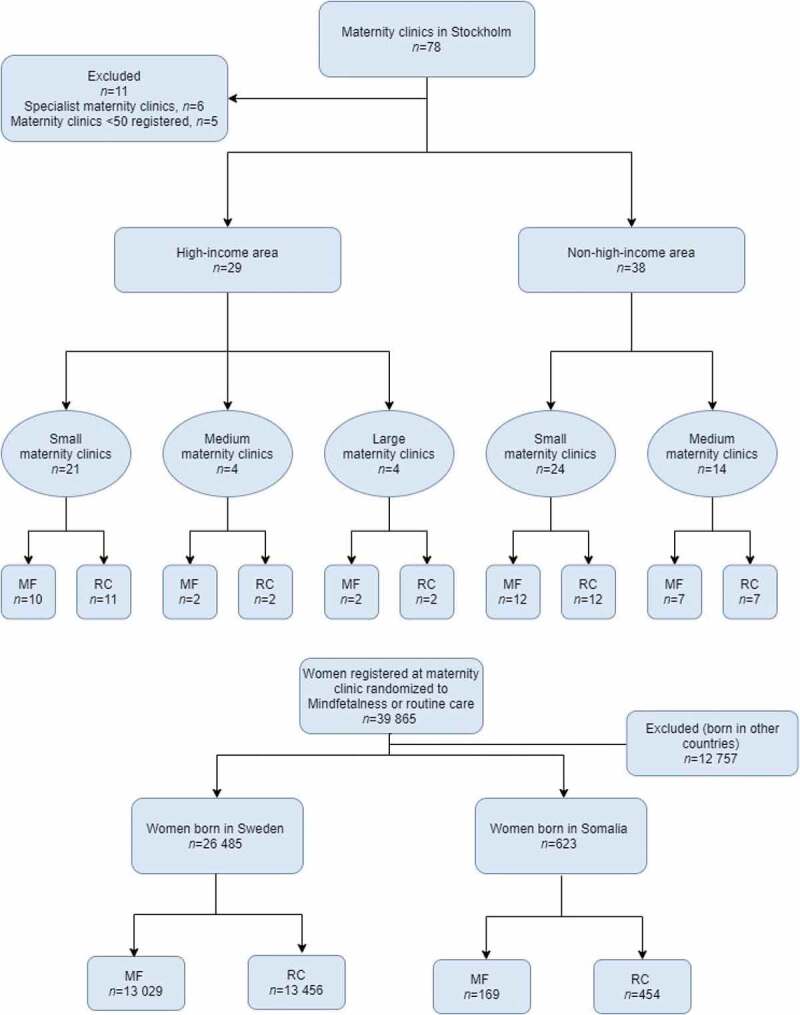
Flow chart.

The randomization was carried out via the 78 maternity clinics in the area (cluster randomization) (Figure 1). Before the randomization, five clinics were excluded because of the small number of women registered annually (<50) and, additionally, six specialized maternity clinics were excluded. Before the randomization, the maternity clinics were divided into two groups based on the socio-demographics of the area in which the clinics were located; high-income areas and non-high-income areas. The clinics were further divided based on the number of women registered at each clinic in 2015, the year before recruitment started: small (*n* < 500), medium (*n* = 500–1000), or large (*n* > 1000). In the end, 33 maternity clinics were randomized to the intervention to promote the proactivity of the pregnant women to practice daily self-monitoring of fetal movements. The method for self-monitoring we introduced is Mindfetalness [17]. In short, the method is practiced daily for about 15 min when the fetus is awake, the woman lies down on her left side and monitors the strength, character, and frequency of the fetal movements but is told not to count each movement. The proactivity mainly comprises the midwife handing out a leaflet (Appendix), and the leaflets given to Somali-born women were written in Somali. No other adaption for their special needs or wants was made, however. Another 34 maternity clinics were allocated to routine care. After the randomization procedure had been completed, due to amalgamations, three maternity clinics merged into one clinic and two maternity clinics merged into another clinic, which resulted in 31 maternity clinics in the Routine-care group. The first woman recruited to the study received a leaflet on 31 August 2016 and the last on 31 January 2018. The women recruited were observed until they gave birth and the last woman included in the study gave birth on 8 June 2018 [16].

In the analysis, we retrieved information about all pregnant women registered at one of the 33 maternal clinics between, and including, 1 November 2016 and 31 January 2018. The observation period for pregnancy outcomes was specific to each woman and newborn. We used intention-to-treat analysis. The primary endpoint, an Apgar score of zero to six (with stillbirth counting as zero) in the newborn, was assessed 5 min after the delivery. As secondary endpoints, we studied an Apgar score of below four in newborns at 5 min, transfer to neonatal intensive care unit (NICU), small for gestational age (weight less or equal to the 10th centile for the gestational age) [19,20], mode of delivery, labor from gestation 41 + 6, and preterm delivery. Most secondary endpoints were observed adjacent to the delivery. Further, the secondary endpoint ‘Contacting health care due to decreased fetal movements’ was based on diagnostic coding according to ICD-10 [21] ‘Examination of decreased fetal movements’ (AM041), where no signs of a compromised fetus and no intervention are documented. The outcome ‘death of the newborn within 27 days after birth’ was also investigated, but no cases were reported.

Data were obtained from a population-based quality register [22] and the planning of the study included two pilot studies [23,24]. The study was registered on 12 August 2016 at www.ClinicalTrials.gov, number NCT02865759.

### Statistical analysis

This is a subgroup analysis of pregnant women born in Somalia compared to women born in Sweden [16,18]. As a metric for the association, we calculated percentage ratios, which are cited as a rate ratio (RR) or adjusted rate ratio (aRR). We employed a log-binomial regression model to adjust the ratio for possible confounding factors and to calculate 95% confidence intervals. The background factors included in the study are standard questions posed to pregnant women, asked by the midwife on registration with maternity care, to plan future care for the woman. In the register, civic status is divided into three alternatives; cohabiting with becoming-father, or single, or, if the woman does not belong to any of these alternatives, she falls into the ‘other family situation’ category. Further, maternal diseases were included in the women’s background information for further exploration if there were large differences between the groups that should be considered when adjusting for possible confounding factors. The possible confounding factors that were considered are comprised age, education level, parity, previous stillbirth, tobacco use at registration, body mass index, assisted reproduction, and maternal diseases. Missing values in the education category were treated as a separate category in the regression model. We used register-based data only.

## Results

Before start of the intervention, one maternity clinic randomized to Mindfetalness declined participation. Due to the intention-to-treat model, women registered at that maternity clinic were included in the Mindfetalness group in the analysis. Approximately 15 500 leaflets were distributed by the midwives. The data we received comprised 39 865 women with singleton pregnancies, who gave birth from gestational week 32 + 0, where 26 485 women were born in Sweden and 623 women were born in Somalia. Among women randomized to intervention with Mindfetalness, 13 029 women were born in Sweden and 169 women were born in Somalia. Corresponding figures for the Routine-care group were 13 456 women born in Sweden and 454 women born in Somalia. The results of the randomization process are shown in Figure 1.

### Women born in Somalia compared to women born in Sweden

In Table 1, background factors are shown for women born in Somalia and women born in Sweden, and the two groups differ generally in background factors.

**Table 1.** Characteristics of 623 women born in Somalia and 26 485 women born in Sweden with a singleton pregnancy, with birth from 32 weeks’ gestation.

	Women born in Somalia *n* (%)	Women born in Sweden *n* (%)	*p*−value
**Age**			
≤24	106 (17.0)	1526 (5.8)	<0.001
25–29	159 (25.5)	6743 (25.5)	0.96
30–34	205 (32.9)	10 525 (39.7)	<0.001
≥35	153 (24.6)	7691 (29.0)	0.02
Education level*			
Shorter than 9 years	171 (27.4)	41 (0.2)	<0.001
Elementary school	113 (18.1)	574 (2.2)	<0.001
Highschool	196 (31.5)	6402 (24.2)	<0.001
University	63 (10.1)	17 874 (67.5)	<0.001
Parity**			
Primipara	131 (21.0)	12 247 (46.2)	<0.001
Multipara	490 (78.7)	14 126 (53.3)	<0.001
**Previous stillbirth**	9 (1.4)	104 (0.4)	0.001
Tobacco user at registration at the maternity clinic**	8 (1.3)	890 (3.4)	0.002
Civic status**			
Cohabiting with becoming father	457 (73.4)	24 669 (93.1)	<0.001
Single	33 (5.3)	349 (1.3)	<0.001
Other family situation	119 (19.1)	849 (3.2)	<0.001
**Body Mass Index^ǂ^**			
<18.5	21 (3.4)	637 (2.4)	0.15
18.5–24.9	175 (28.1)	16 770 (63.3)	<0.001
25.0–29.9	216 (34.7)	5465 (20.6)	<0.001
30.0–34.9	137 (22.0)	1743 (6.6)	<0.001
≥35.0	57 (9.1)	662 (2.5)	<0.001
Assisted reproduction**	6 (1.0)	1686 (6.4)	<0.001
**Maternal diseases**			
Diabetes mellitus	1 (0.2)	33 (0.1)	0.55
Coronary heart disease	5 (0.8)	438 (1.7)	0.11
Thrombosis	6 (1.0)	235 (0.9)	0.83
Systemic lupus erythematosus (SLE)	0 (0.0)	44 (0.2)	0.63
Psychiatric care	15 (2.4)	4281 (16.2)	<0.001
Endocrine disease	30 (4.8)	1852 (7.0)	0.04
Epilepsy	2 (0.3)	137 (0.5)	0.77
Chronic hypertension	3 (0.5)	130 (0.5)	1.00
Other disease	23 (3.7)	2772 (10.5)	<0.001
Medication or psychological treatment for mental illness	7 (1.1)	1716 (6.5)	<0.001

*Missing: Women born in Somalia *n* = 80 (12.8%), women born in Sweden *n* = 1594 (6.0%)

**Missing: Women born in Somalia *n* = 2 (0.3%), women born in Sweden *n* = 112 (0.4%)

^ǂ^Missing: Women born in Somalia *n* = 17 (2.7%), women born in Sweden *n* = 1208 (4.6%)

**Figure 2.**
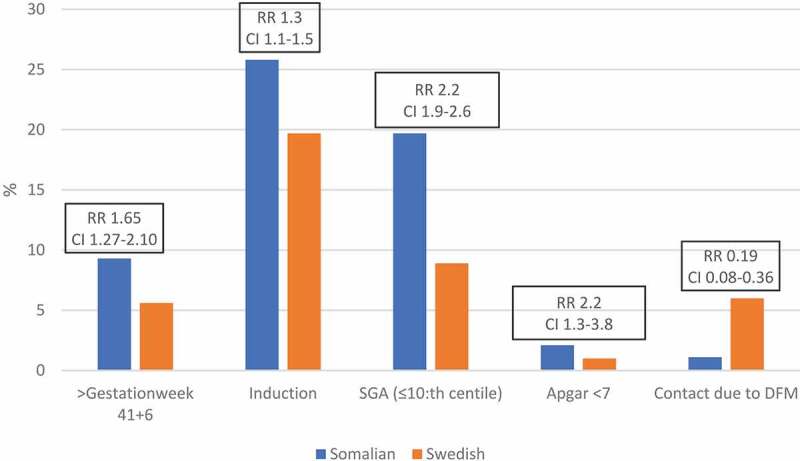
A comparison in obstetric and birth outcomes between women from Somalia and Sweden.

Compared to women born in Sweden, women born in Somalia had a higher risk of adverse pregnancy outcomes (Figure 2). Women born in Somalia had a higher risk of their babies having an Apgar score of less than seven at 5 min (RR 2.17, CI 1.25–3.77, *p*-value 0.01) and having a baby that was small for gestational age (≤10th centile: RR 2.22, CI 1.88–2.61, *p*-value <0.001). Further, women from Somalia had a higher risk of stillbirth (RR 6.86, CI 2.68–17.58, *p*-value 0.001) (data not shown in table). More babies were born after 41 + 6 weeks’ gestation among women born in Somalia (RR 1.65, CI 1.27–2.10, *p*-value <0.001). Women from Somalia sought care due to decreased fetal movements to a lower extent than did women from Sweden (1.1% versus 6.0%, RR 0.19, CI 0.08–0.36, *p*-value <0.001). As shown in Table S1, the statistically significant differences between women born in Somalia and Sweden above remain after adjustment for potential confounders.

Labor induction was more common for women born in Somalia compared to women born in Sweden (25.8% versus 19.7%, RR 1.3, CI 1.1–1.5, *p*-value <0.001) and when adjusting for potential confounders, one single variable at a time, the difference remained, except for BMI at registration (Table S1). The percentage of cesarean sections among women born in Somalia was lower than women born in Sweden, 16.7% versus 19.2%, but the percentage of emergency cesarean sections was higher among women born in Somalia (10.1% versus 8.8%) (Table S1).

### Women born in Somalia, Mindfetalness compared to routine care

Characteristics for women born in Somalia are displayed in Table 2, where the Mindfetalness group differed in age and educational level compared to the Routine-care group.

**Table 2.** Characteristics of women born in Somalia with singleton pregnancy, 169 registered at a maternity clinic randomized to Mindfetalness, and 454 women registered at a maternity clinic randomized to routine care.

	Mindfetalness *n* (%)	Routine care *n* (%)	*p*-value
**Age**			
≤24	41 (24.3)	65 (14.3)	0.006
25–29	40 (23.7)	119 (26.2)	0.54
30–34	53 (31.4)	152 (33.5)	0.63
≥35	35 (20.7)	118 (26.0)	0.21
Education level*			
Shorter than 9 years	33 (19.5)	138 (30.4)	0.007
Elementary school	27 (16.0)	86 (18.9)	0.42
Highschool	58 (34.3)	138 (30.4)	0.38
University	18 (10.7)	45 (9.9)	0.77
Parity**			
Primipara	39 (23.1)	92 (20.3)	0.44
Multipara	129 (76.3)	361 (79.5)	0.38
**Previous stillbirth**	2 (1.2)	7 (1.5)	1.00
Tobacco user at registration**	3 (1.8)	5 (1.1)	0.45
**Civic status**			
Cohabiting with becoming father	120 (71.0)	337 (74.2)	0.42
Single	15 (8.9)	18 (4.0)	0.02
Other family situation	27 (16.0)	92 (20.3)	0.25
**Body Mass Index^ǂ^**			
<18.5	9 (5.3)	12 (2.6)	0.13
18.5–24.9	51 (30.2)	124 (27.3)	0.48
25.0–29.9	49 (29.0)	167 (36.8)	0.07
30.0–34.9	36 (21.3)	101 (22.2)	0.83
≥35.0	14 (8.3)	43 (9.5)	0.76
Assisted reproduction**	1 (0.6)	5 (1.1)	1.00
**Maternal diseases**			
Diabetes mellitus	0 (0)	1 (0.2)	1.00
Coronary heart disease	2 (1.2)	3 (0.7)	0.62
Thrombosis	1 (0.6)	5 (1.1)	1.00
Systemic lupus erythematosus (SLE)	0 (0)	0 (0)	-
Psychiatric care	4 (2.4)	11 (2.4)	1.00
Endocrine disease	7 (4.1)	23 (5.1)	0.83
Epilepsy	1 (0.6)	1 (0.2)	0.47
Chronic hypertension	0 (0)	3 (0.7)	0.57
Other disease	4 (2.4)	19 (4.2)	0.35
Medication or psychological treatment for mental illness	2 (1.2)	5 (1.1)	1.00

*Missing: Mindfetalness *n* = 33 (19.5%); Routine care *n* = 47 (10.4%)

**Missing: Mindfetalness *n* = 1 (0.6%); Routine care *n* = 1 (0.2%)

^ǂ^Missing: Mindfetalness *n* = 10 (5.9%); Routine care *n* = 7 (1.5%)

As displayed in Table 3, among women born in Somalia, in comparison with routine care, no statistically significant differences were seen in Apgar score less than seven at 5 min (0.6% versus 2.7%, aRR 0.22, CI 0.01–1.11) and babies in need of transfer to NICU (4.7% versus 7.5%, aRR 0.54, CI 0.24–1.08). Women from Somalia in the Mindfetalness group had preterm labor to a lower extent than women in the Routine-care group (0.6% versus 3.1%, aRR 0.15, CI 0.01–0.75).

**Table 3.** Obstetric outcome from gestation 32 + 0 among 169 Somali women with singleton pregnancy registered at a maternity clinic randomized to Mindfetalness and 454 Somali women with singleton pregnancy registered at a maternity clinic randomized to routine care.

Outcome	Mindfetalness *n* (%)	Routine care *n* (%)	Rate Ratio (95% CI)	*p*-value	Adjusted^†^ Rate Ratio (95% CI)	Adjusted^†^*p*-value
Spontaneous start of labour	106 (62.7)	315 (69.4)	0.90 (0.79–1.02)	0.12	0.89 (0.77–1.01)	0.08
Induction of labour	53 (31.4)	108 (23.8)	1.32 (0.99–1.73)	0.06	1.32 (0.98–1.74)	0.07
Cesarean section (total)	26 (15.4)	78 (17.2)	0.90 (0.58–1.32)	0.59	0.91 (0.59–1.35)	0.64
Pre-labour	10 (5.9)	31 (6.8)	0.87 (0.41–1.66)	0.68	0.91 (0.43–1.75)	0.78
In labour	16 (9.5)	47 (10.4)	0.91 (0.52–1.53)	0.74	0.90 (0.51–1.52)	0.71
Preterm delivery (<37 + 0)	1 (0.6)	14 (3.1)	0.19 (0.01–0.94)	0.04	0.15 (0.01-–0.75)	0.02
Birth gestation >41 + 6	20 (11.8)	38 (8.4)	1.41 (0.83–2.33)	0.20	1.37 (0.80–2.28)	0.25
Apgar Score <7 at 5 min*^‡^	1 (0.6)	12 (2.7)	0.22 (0.01–1.12)	0.07	0.22 (0.01–1.11)	0.07
Apgar Score <4 at 5 min*^‡^	0 (0)	7 (1.5)		0.20^¶^	NA	
Stillbirth	0 (0.0)	5 (1.1)		0.33^¶^	NA	
Small for gestational age**^∫^	36 (21.3)	87 (19.2)	1.11 (0.77–1.55)	0.56	1.08 (0.75–1.52)	0.67
Admitted to NICU	8 (4.7)	34 (7.5)	0.63 (0.28–1.27)	0.21	0.54 (0.24–1.08)	0.08

^†^Adjusted for Age, Educational level (missing as one category)

**^‡^**Missing *n* = 0 (0%) Mindfetalness, *n* = 3 (0.7%) Routine care

^¶^Fishers exact test

^∫^Missing *n* = 0 (0%) Mindfetalness, *n* = 1 (0.2%) Routine care

*Stillbirth = Apgar score 0

**≤10th centile for the gestational age

NICU = Neonatal intensive care unit

Women born in Somalia in the Mindfetalness group had a lower rate of spontaneous start of labor (62.7% versus 69.4%, aRR 0.89, CI 0.77–1.01) and a higher rate of labor induction (31.4% versus 23.8%, aRR 1.32, CI 0.98–1.74) than those in the Routine-care group. A higher percentage of women born in Somalia in the Mindfetalness group gave birth after gestation 41 + 6 (11.4% versus 8.4%) and few women had their babies transferred to NICU (4.7% vs. 7.5%, aRR 0.63, CI 0.24–1.08). The percentage of women born in Somalia who contacted health care due to decreased fetal movements was 1.8% (*n* = 3) in the Mindfetalness group and 0.9% (*n* = 4) in the Routine-care group (not in table).

## Discussion

We have studied 623 women born in Somalia and 26 485 women born in Sweden out of 39 865 women in a cluster randomized controlled trial, being encouraged to monitor fetal movements daily (Mindfetalness group) or not (Routine care-group). Women born in Somalia, as compared to the women born in Sweden, gave birth to a baby with an Apgar score of less than seven at 5 min more frequently. A large difference between the two groups was also found for the percentage of children born small for gestational age. Further, women born in Somalia had labor after gestation week 41 + 6 to a higher extent. We also found that Somali-born women contacted obstetric care for decreased fetal movements to a much lesser extent than Swedish-born women. The differences were somewhat larger among women randomized to routine care as compared to women allocated to receiving proactivity concerning self-monitoring of fetal movements.

The risk ratio for Apgar score of less than seven at 5 min and stillbirth was higher for children to women born in Somalia as compared to children to women born in Sweden. We found that the number of babies born small for gestational age in this setting (less or equal to the 10th centile) was higher among women from Somalia (19.7% or 6.3%), compared to women born in Sweden, as previously reported [25]. Despite existing scientific knowledge, so far, Swedish health care has not managed to reduce the large differences between women from Somalia and Sweden. Severe vitamin D deficiency is common among pregnant women from Somalia, but the obstetrical consequences are unknown [26,27]. However, in Stockholm, Sweden, local guidelines for the maternity clinics suggest that extra attention is given to test for possible vitamin D deficiency among women born outside Sweden [28]. Recently, researchers found Vitamin D deficiency among 73% of women from Somalia, living in Sweden, but the comorbidity was low [29]. Worth noting is that 65.8% of the women from Somalia were overweight/obese (Swedish women 29.7%). This corresponds to a recent study from Finland showing that 63.8% of women born in Somalia were overweight/obese compared to 29.7% of Finnish women [30]. One study found an association between obesity (but not overweight) and higher risk for giving birth to a baby small for gestational age (RR 2.66, CI 2.01, 3.52) [31] but other studies have shown inconsistent results of the link between overweight and SGA [32]. Another possible explanation is that placenta-mediated diseases (like preeclampsia and SGA) and epigenetic factors can be transferred to the following generations [33]. The risk for having an SGA baby increases by almost three times, if the mother has an SGA background [34].

Concerning potentially avoidable perinatal deaths, an author group suggested women from the horn of Africa have a higher risk (OR 6.2) compared to women born in Sweden [35]. Suggested mechanisms include delay in seeking health care for pregnancy complications or decreased fetal movements, inadequate medication, insufficient surveillance of intrauterine growth restriction, misinterpreted cardiotocography, refusing an appropriate cesarean section, and interpersonal miscommunication. Rassjo and co-authors found that women born in Somalia, as compared to being born in Sweden, register later at a maternal clinic when pregnant and make less visits during pregnancy [25]. In Norway [5], Saastad and co-authors found that non-western women had increased risk of receiving sub-standard care (OR 2.4, CI 1.5–3.9) and more often received sub-standard obstetrical care. Additionally, inadequate communication was documented in 47% of non-western mothers.

When interviewing 15 women from Somalia, researchers found that some women had fear of cesarean section and had reduced food intake when pregnant to have smaller babies for reducing the risk of cesarean section [36]. In our study, the percentage of emergency cesarean sections among women born in Somalia was higher than that among women born in Sweden; a similar result has been seen in Denmark [37]. Essen et al. [36] identified some explanations for the increased risk of a worse pregnancy outcome among Somali-born women. They enumerated women from Somalia may only see a doctor if it is ‘really necessary’ and that the baby is a gift from God and only God knows whether something is wrong with the pregnancy. The ongoing Hooyo-project [10] has an interesting approach, investigating the effects of group antenatal care among women from Somalia, giving birth in Sweden. We do not know whether that project included providing information to the mothers about fetal movements; however, this is probably the case as information about fetal movements should be included in antenatal care [38]. The targeted women may have similar needs and it may be easier to reach them in this context. This in turn can raise their knowledge, understanding, and lead to improve pregnancy outcomes.

Women born in Sweden, registered at a maternity clinic in the capital Stockholm, might not be representative for women born in Sweden in general. In Stockholm, the women have higher education level, lower BMI, and are older when giving birth, compared to pregnant women in Sweden in general. The differences in background factors between the two groups might be less in other parts of Sweden and are important to consider when analyzing the differences in outcomes, as these factors are potential confounders, which might affect the risk ratio.

We observed that the number of times women born in Somalia contacted health care due to decreased fetal movements was lower than among the women born in Sweden. In total, 6.0% of women born in Sweden and 1.1% among women born in Somalia sought care due to decreased fetal movements. In earlier international studies, 6–15% of all pregnant women contacted health care due to decreased fetal movements [39–41] and, in Stockholm, Sweden, this figure is 9.3% (from 28 weeks’ gestation) [42]. Based on seven Somali-born women only, those who were randomized to Mindfetalness sought care due to decreased fetal movements more often than those randomized to routine care. We do not know if the difference depends on random variation or reflects a true effect of Mindfetalness. The leaflet included instructions to the pregnant women to contact health care if they felt concerned about the unborn baby. Few women in general contacted health care and the result is difficult to draw conclusions from, but being provided with written information in their own language might have facilitated and contributed to their decision to contact health care. When women contact health care due to decreased fetal movements, the health-care professionals are able to judge whether extended examinations are needed, and are given the opportunity to discover, for example, small-for-gestational-age babies, so necessary interventions such as labor induction are made in time.

The rate of preterm labor, babies having an Apgar score below seven at 5 min, and babies transferred to NICU was lower in the Mindfetalness group than in the Routine-care group. However, the compared groups were unbalanced, women in the Mindfetalness group had a higher education level, most women were single and below 24 years of age. These potential confounders might have affected the results, but almost no differences are seen after adjustment for age and education level. The risk of false-positive findings is greater in subgroup analysis [43]. However, the study did not have statistical power to address the hypothesis that Mindfetalness reduces the number of babies with an Apgar score of less than seven at 5 min or babies in need of transfer to NICU. This is important to consider when analyzing the sub-study results. The effect of Mindfetalness in reducing preterm labor is unknown, and the effect was not observed in the Mindfetalness trial for the whole group [16]. Random fluctuation, or an unbalanced distribution of confounding factors not accounted for in the adjustments, may offer an explanation for the modifications seen rather than a true effect.

### Conclusion

Large differences in pregnancy outcomes persist between Somali-born women and Swedish-born women giving birth in Stockholm. The higher risk of having a baby with a low Apgar score and giving birth to a baby small for gestational age needs to be further investigated. Reasonably, we can infer that there are needs among Somali-born women that are not being met. We also observed that Somali-born women seek obstetric care for decreased fetal movements to a lesser degree than do Swedish-born women. We speculate that encouraging proactivity in the self-monitoring of fetal movements (Mindfetalness) may be particularly helpful for Somali-born women. The results do not provide conclusive evidence that this is the case; we need more data before definitive conclusions can be drawn. By conducting focus groups and interviewing women born in Somalia, living in Sweden, a culturally adapted Mindfetalness leaflet can be designed and evaluated in a new trial that includes only Somali-born women, allocated to either Mindfetalness or routine care.

## Supplementary Material

Supplemental Material

## Paper context

Migrating women have worse pregnancy outcomes than receiving-country nationals.

Somali-born women have a higher risk of stillbirth and having small-for-gestational-age infants and they contacted health care due to decreased fetal movements to a lesser extent than Swedish-born women. The differences found were lower among women allocated to observe fetal movements daily towards the end of pregnancy.

Self-monitoring of fetal movements can be one way forward for diminishing the differences in outcomes between Somali-born and Swedish-born women.

## Appendix Mindfetalness leaflet

**Mindfetalness – a method for focusing upon fetal movements**

The first perception of fetal movements is sometimes described as a gentle tickling. As the pregnancy proceeds and the fetus develops the movements become more distinct. Just as new-born babies vary, there are differences between fetuses. Some fetuses are very active during their time in the uterus whilst others are calm, but all fetuses move up until birth. Each unborn baby has its own pattern of movements and at the end of the pregnancy the pattern can be recognized. The fetus moves between periods of wakefulness with much movement and calmer periods of rest. Movement frequency usually peaks around week 32 of pregnancy and remains for the most part at that level until delivery. The movements increase in strength as the fetus grows but at the end of the pregnancy can be experienced differently to the movements felt earlier.

That the fetus moves is a good sign and many pregnant women describe how they notice their unborn baby’s movements every day. One systematic way of observing the movements is the method of *Mindfetalness* that can be used to get to know the movement pattern. An appropriate time to begin with *Mindfetalness* is in gestational week 28.

**How to put Mindfetalness into practice?**

Mindfetalness is practiced ideally on a daily basis. **Choose a time of day that suits you best but also wait until you feel that your unborn baby is having a period of wakefulness**. Lie down or sit comfortably when you engage in Mindfetalness. If you lie down, then preferably on your left side. The movements are felt more distinctly when you lie down, and the blood flow is at its best in the uterus on the left side, which is good for the fetus. Concentrate on your unborn baby’s movements for approximately 15 minutes. You will feel yourself if you need a longer or shorter time to perceive the movements. For some women it is enough just to get an idea of how the movements feel, others prefer to write notes about what they experience. At the end of this information, there is space in which you can write down how you experience the movements. If you have a smartphone or computer you can write your impressions at www.mindfetalness.com

*During Mindfetalness you focus upon*:

The intensity of the movements

The way in which the baby moves

How much the baby moves

*The questions to be answered are*:

Can the movements be felt distinctly?

Are the movements of the same intensity as usual?

Does the fetus move as much as usual?

**Fetal movements at the end of the pregnancy**

The unborn baby’s movements can be divided into two main groups: large movements and small movements. The large movements are felt distinctly; this can be when the fetus kicks or stretches out its body. The small movements that the fetus makes, but which are not felt, are gripping movements with fingers and toes as well as breathing movements. In approximately weeks 25 to 30 the movements begin to become organized and the unborn baby has periods of wakefulness interspersed with periods of rest lasting approximately 40 minutes up to an hour.

The movements change as the pregnancy proceeds and can feel different due to the fact that the space the fetus has at its disposal becomes smaller, although this does not affect the frequency of the movements. Women at full-term pregnancy often describe how the movements feel powerful, pushing, stretching, large, from side to side, slow, and light.

If you focus upon the fetal movements for a while every day when the fetus has a period of wakefulness, you can gain a good understanding of your unborn baby’s movement pattern. It is important the observation occurs when the fetus is awake (the fetus is less active during a rest period). There can be wide variations from fetus to fetus regarding the frequency and intensity of the movements.

Women who have tried *Mindfetalness* describe how they felt calm, present, and focussed while using the method. They also describe the period as a communication with their unborn baby and that they experienced a powerful bonding with their baby. Only you can decide whether the method suits you.

**Summary**

The movements become organized during pregnancy weeks 25 to 30 and the fetus has periods of wakefulness interspersed with periods of rest, approximately 40 minutes up to an hour. Most fetuses have, at the end of the pregnancy, a daily rhythm and are active in the evening.

In pregnancy week 32 a plateau phase is reported regarding the frequency of movements but there is nothing to indicate that the movements decrease at the end of pregnancy.

There can be a wide variation between fetuses in the frequency and intensity of their movements.

Get to know your unborn baby’s movement pattern during pregnancy. Trust your intuition.

If you are concerned that the fetus is moving less or that the movements are weaker, you should contact health care.

**Questions and answers about fetal movements**

**What does the fetus do in the uterus?**

As the fetus grows, the movements become more distinct and successively regular. Small movements are not felt, e.g. when the fetus sucks its thumb or flexes its toes. Kicks, and when the child stretches out, can usually be felt clearly and many also feel when the child hiccups (small rhythmic jerks) during the latter part of pregnancy.

During the final months of the pregnancy, the movements are distinct and powerful, but maybe experienced as being of a different character compared with when the fetus had a larger space at its disposal. Some women describe how the unborn baby stretches out, as if the fetus is trying to stretch as the space begins to be tight. Others describe the movements as large that they involve the unborn baby’s whole body and can be described as slow. The larger the fetus the more distinct the movement.

**Is it true that the fetus moves less towards the end of pregnancy?**

No, this is not true. Fetal movements increase up until pregnancy week 32; thereafter, and up until delivery, the frequency of movements generally remains the same. It is important to remember that the fetus should continue to be active throughout the pregnancy.

**Does the fetus move the whole time?**

The fetus does not move the whole time. All unborn babies are calm and sleep for short periods. There can be wide variations from fetus to fetus regarding the frequency and intensity of the movements.

**Can it be more difficult for some women to feel the movements?**

It is probable that it is easier to feel the movements if the woman lies on her left side and concentrates on them. Some women describe how, in spite of doing so, they have great difficulty in feeling their unborn baby move. Extreme overweight, amongst other things, can make it more difficult. If one is much stressed, it may be more difficult to feel the movements.

**What should I do if I feel that the movements become fewer towards the end of the pregnancy?**

If the movements decrease in intensity or frequency and deviate from the fetus’ normal way of moving, it can be a sign that the child is not doing so well in the uterus. Most pregnant women who experience fewer and weaker movements give birth to a healthy child, but there is an increased risk that the fetus is not fit. If you experience that the movements have become fewer and weaker, and you feel that there is a difference compared with earlier in the pregnancy it should *not* be interpreted as something normal until the child has been examined.

**Diary of my unborn baby’s activities**

**Table:**

**Pregnancy week** **28+**	**How my unborn baby moved**

**Pregnancy week** **29+**	**How my unborn baby moved**

**Pregnancy week** **30+**	**How my unborn baby moved**

**Pregnancy week** **31+**	**How my unborn baby moved**

**Diary of my unborn baby’s activities**

**Table:**

**Pregnancy week** **32+**	**How my unborn baby moved**

**Pregnancy week** **33+**	**How my unborn baby moved**

**Pregnancy week** **34+**	**How my unborn baby moved**

**Pregnancy week** **35+**	**How my unborn baby moved**

**Diary of my unborn baby’s activities**

**Table:**

**Week of pregnancy** **36+**	**How my unborn baby moved**

**Week of pregnancy** **37+**	**How my unborn baby moved**

**Week of pregnancy** **38+**	**How my unborn baby moved**

**Week of pregnancy** **39+**	**How my unborn baby moved**

**Diary of my unborn baby’s activities**

**Table:**

**Week of pregnancy** **40+**	**How my unborn baby moved**

**Week of pregnancy** **41+**	**How my unborn baby moved**

**Week of pregnancy** **42+**	**How my unborn baby moved**
