# Supplementary material for: Pregnancy outcomes among women born in Somalia and Sweden giving birth in the Stockholm area – a population-based study
Source: Glob Health Action. 2020 Aug 3;13(1):1794107. doi: 10.1080/16549716.2020.1794107 (PMC7480426; doi:10.1080/16549716.2020.1794107)
Supplement: Supplemental Material [file ZGHA_A_1794107_SM8055.docx]

**Supplementary table 1**. Obstetric and birth outcomes among women with singleton pregnancies with birth from 32 weeks’ gestation. Comparison between 623 women born in Somalia and 26 485 women born in Sweden. Obstetric outcomes adjusted for tobacco use at registration, age, educational level, BMI, parity and previous stillborn child.

| Outcome | Somalian  *n* (%) | Swedish  *n* (%) | RR | *p-*value | CI |
| --- | --- | --- | --- | --- | --- |
| Spontaneous start of labour | 421 (67.6) | 18 501 (69.9) | 0.97 | 0.22 | 0.91−1.02 |
| Adjusted for: |  |  |  |  |  |
| Tobacco at registration |  |  | 0.97 | 0.19 | 0.91−1.02 |
| Age |  |  | 0.96 | 0.09 | 0.90−1.01 |
| Educational level |  |  | 0.98 | 0.59 | 0.92−1.04 |
| BMI at registration |  |  | 1.01 | 0.64 | 0.96−1.07 |
| Parity |  |  | 0.97 | 0.22 | 0.91−1.02 |
| Previous stillborn child |  |  | 0.97 | 0.29 | 0.92−1.02 |
| Induction of labour | 161 (25.8) | 5229 (19.7) | 1.31 | <0.001 | 1.14−1.49 |
| Adjusted for: |  |  |  |  |  |
| Tobacco at registration |  |  | 1.32 | <0.001 | 1.15−1.50 |
| Age |  |  | 1.30 | <0.001 | 1.13−1.48 |
| Educational level |  |  | 1.20 | 0.03 | 1.02−1.40 |
| BMI at registration |  |  | 1.11 | 0.14 | 0.96−1.27 |
| Parity |  |  | 1.44 | <0.001 | 1.25−1.65 |
| Previous stillborn child |  |  | 1.29 | <0.001 | 1.12−1.47 |
| Cesarean section | 104 (16.7) | 5086 (19.2) | 0.87 | 0.11 | 0.72−1.03 |
| Adjusted for: |  |  |  |  |  |
| Tobacco at registration |  |  | 0.87 | 0.12 | 0.73−1.03 |
| Age |  |  | 0.90 | 0.23 | 0.75−1.07 |
| Educational level |  |  | 0.92 | 0.40 | 0.75−1.11 |
| BMI at registration |  |  | 0.77 | 0.002 | 0.64−0.91 |
| Parity |  |  | 0.89 | 0.18 | 0.74−1.05 |
| Previous stillborn child |  |  | 0.86 | 0.08 | 0.72−1.02 |
| Cesarean pre-labour | 41 (6.6) | 2767 (10.4) | 0.63 | <0.001 | 0.46−0.84 |
| Adjusted for: |  |  |  |  |  |
| Tobacco at registration |  |  | 0.63 | <0.001 | 0.46−0.84 |
| Age |  |  | 0.68 | 0.006 | 0.50−0.90 |
| Educational level |  |  | 0.68 | 0.02 | 0.48−0.93 |
| BMI at registration |  |  | 0.61 | <0.001 | 0.44−0.81 |
| Parity |  |  | 0.55 | <0.001 | 0.40−0.73 |
| Previous stillborn child |  |  | 0.62 | <0.001 | 0.45−0.82 |
| Cesarean in labour | 63 (10.1) | 2319 (8.8) | 1.15 | 0.25 | 0.90−1.45 |
| Adjusted for: |  |  |  |  |  |
| Tobacco at registration |  |  | 1.16 | 0.23 | 0.91−1.46 |
| Age |  |  | 1.16 | 0.23 | 0.90−1.46 |
| Educational level |  |  | 1.19 | 0.22 | 0.90−1.54 |
| BMI at registration |  |  | 0.93 | 0.55 | 0.72−1.17 |
| Parity |  |  | 1.49 | 0.002 | 1.17−1.87 |
| Previous stillborn child |  |  | 1.15 | 0.25 | 0.90−1.45 |
| Preterm delivery | 15 (2.4) | 962 (3.6) | 0.66 | 0.09 | 0.38−1.05 |
| Adjusted for: |  |  |  |  |  |
| Tobacco at registration |  |  | 0.67 | 0.09 | 0.38−1.06 |
| Age |  |  | 0.64 | 0.06 | 0.37−1.02 |
| Educational level |  |  | 0.63 | 0.08 | 0.35−1.06 |
| BMI at registration |  |  | 0.59 | 0.03 | 0.33−0.95 |
| Parity |  |  | 0.73 | 0.21 | 0.42−1.17 |
| Previous stillborn child |  |  | 0.66 | 0.08 | 0.38−1.05 |
| Gestation >41+6 | 58 (6.3) | 675 (2.6) | 1.65 | <0.001 | 1.27−2.10 |
| Adjusted for: |  |  |  |  |  |
| Tobacco at registration |  |  | 1.65 | <0.001 | 1.27−2.10 |
| Age |  |  | 1.67 | <0.001 | 1.28−2.12 |
| Educational level |  |  | 1.69 | 0.001 | 1.24−2.24 |
| BMI at registration |  |  | 1.62 | <0.001 | 1.24−2.08 |
| Parity |  |  | 1.97 | <0.001 | 1.51−2.50 |
| Previous stillborn child |  |  | 1.66 | <0.001 | 1.28−2.11 |
| Apgar score less than 7 at 5 minutes‡ | 13 (2.1) | 255 (1.0) | 2.17 | 0.01 | 1.19−3.61 |
| Adjusted for: |  |  |  |  |  |
| Tobacco at registration |  |  | 2.19 | 0.01 | 1.19−3.64 |
| Age |  |  | 2.08 | 0.02 | 1.14−3.48 |
| Educational level |  |  | 2.42 | 0.01 | 1.21−4.34 |
| BMI at registration |  |  | 2.03 | 0.03 | 1.10−3.42 |
| Parity |  |  | 2.50 | 0.005 | 1.36−4.17 |
| Previous stillborn child |  |  | 2.16 | 0.01 | 1.18−3.59 |
| Apgar score less than 4 at 5 minutes‡ | 7 (1.1) | 87 (0.3) | 3.43 | 0.008 | 1.45−6.85 |
| Adjusted for: |  |  |  |  |  |
| Tobacco at registration |  |  | 3.41 | 0.008 | 1.44−6.80 |
| Age |  |  | 3.32 | 0.01 | 1.39−6.67 |
| Educational level |  |  | 3.74 | 0.01 | 1.43−8.23 |
| BMI at registration |  |  | 3.69 | 0.006 | 1.53−7.55 |
| Parity |  |  | 3.63 | 0.006 | 1.52−7.32 |
| Previous stillborn child |  |  | 3.39 | 0.008 | 1.43−6.77 |
| Stillbirth | 5 (0.8) | 31 (0.1) | 6.86 | 0.001 | 2.35−16.07 |
| Adjusted for: |  |  |  |  |  |
| Tobacco at registration |  |  | 6.84 | 0.001 | 2.34−16.05 |
| Age |  |  | 6.71 | 0.002 | 2.27−15.92 |
| Educational level |  |  | 5.17 | 0.01 | 1.49−14.20 |
| BMI at registration |  |  | 7.40 | 0.001 | 2.47−18.03 |
| Parity |  |  | 6.96 | 0.001 | 2.35−16.64 |
| Previous stillborn child |  |  | 6.56 | 0.002 | 2.23−15.46 |
| Small for gestational age¶* | 123 (19.8) | (9.0) | 2.19 | <0.001 | 1.85−2.56 |
| Adjusted for: |  |  |  |  |  |
| Tobacco at registration |  |  | 2.20 | <0.001 | 1.86−2.57 |
| Age |  |  | 2.13 | <0.001 | 1.80−2.50 |
| Educational level |  |  | 2.00 | <0.001 | 1.62−2.42 |
| BMI at registration |  |  | 2.34 | <0.001 | 1.96−2.75 |
| Parity |  |  | 2.83 | <0.001 | 2.40−3.29 |
| Previous stillborn child |  |  | 2.19 | <0.001 | 1.85−2.57 |
| Transfer to NICU | 42 (6.7) | 1746 (6.6) | 1.02 | 0.88 | 0.75−1.35 |
| Adjusted for: |  |  |  |  |  |
| Tobacco at registration |  |  | 1.03 | 0.86 | 0.75−1.36 |
| Age |  |  | 1.00 | 0.98 | 0.73−1.32 |
| Educational level |  |  | 1.11 | 0.51 | 0.80−1.51 |
| BMI at registration |  |  | 0.85 | 0.29 | 0.61−1.14 |
| Parity |  |  | 1.13 | 0.42 | 0.83−1.51 |
| Previous stillborn child |  |  | 1.02 | 0.89 | 0.75−1.35 |

‡ Data are missing for 49 women (3 in Mindfetalness group and 46 in Routine care group)

¶ Data are missing for 24 women (1 in Mindfetalness group and 23 in Routine care group)

*≤10^th^ centile for gestational age

NICU Neonatal intensive care unit
